# Congenital Hypertrophic Stenosis of the Pylorus

**Published:** 1904-04-30

**Authors:** 


### CONGENITAL HYPERTROPHIC STENOSIS OF THE PYLORUS.

Of the large number of infants who die annually of marasmus and persistent vomiting, it is probable that a certain proportion are instances of a definite pathological condition which can be satisfactorilly treated by surgical means, though hardly ever amenable to dietetic or medicinal methods. Although this condition was described more than 60 years ago, it has only recently attracted much attention; and, as frequently happens, the increased care expended on its recognition has thrown doubts on its extreme rarity. Anatomically the cases present an immense increase in the circular muscle fibres of the pylorus which appears as a firm cylinder about an inch long, with a minute lumen. The mucous membrane is thrown into folds, still further obstructing the passage, and the stomach is markedly dilated. Thickening of the stomach, duodenum, and œsophagus, of the pyloric submucosa, and very rarely of the longitudinal muscle fibres occasionally occurs. Originating *in utero*, the condition is probably a primary hyperplasia of the pylorus and possibly also of the stomach, duodenum, and œsophagus, with a secondary pyloric spasm producing more or less complete obstruction. The symptoms usually set in a few weeks after birth, and death ensues in most cases at the age of three or four months. A few cases, however, have recovered under careful dieting, and appearances have been found post-mortem in much older children, and even in adults, presumably due to congenital pyloric hyperplasia. In a typical case vomiting is the first symptom; two or three meals are retained in the stomach, and the whole is then rejected. A clean tongue and sweet breath exclude gastritis; the stools are small and infrequent. Wasting, with obvious dilatation of the stomach and visible peristalsis ensue, and sometimes careful palpation can detect the thickened pylorus. Atypical cases are met with in which the obstruction is incomplete or only occasional. In these the progress is intermittent, the child retaining its food and putting on weight perhaps for a fortnight at a time; in some, too, the dilatation of the stomach is slight and the vomiting not distinctive. In all the cardinal diagnostic points are obstinate emesis and the discovery of a pyloric tumour.

In view of the brilliant results obtained by surgical measures—either by pyloroplasty or a modified Loreta's operation—as contrasted with the usually fatal issue of even the milder cases under merely medical treatment, it is highly important that general practitioners, who see by far the greatest number of marasmic infants, should have their attention directed to this condition. A good many cases which might have been saved by operation, undoubtedly, owing to a general want of familiarity with the symptoms and course, escape diagnosis, thus adding their quota to our huge infant mortality.

